# A Comparative Study Between Video Laryngoscope and Video Stylet for Tracheal Intubation in Patients With Simulated Cervical Fracture Injury: A Prospective Randomised Controlled Study

**DOI:** 10.7759/cureus.66360

**Published:** 2024-08-07

**Authors:** Ajin Sanu, Syed Moied Ahmed

**Affiliations:** 1 Anaesthesiology, Jawaharlal Nehru Medical College Hospital, Aligarh Muslim University, Aligarh, IND; 2 Anaesthesiology and Critical Care, Jawaharlal Nehru Medical College, Aligarh Muslim University, Aligarh, IND

**Keywords:** randomised controlled trial, cmac video stylet, comparison of video stylet and video laryngoscope, simulated cervical fracture injury, karl storz video stylet

## Abstract

Purpose

Video laryngoscopes were being used more often in cases of potentially difficult airways. The Karl Storz video stylet offered clear advantages over conventional laryngoscopes for patients with cervical spine fractures. This study aimed to compare the performance of the C-MAC video laryngoscope with the new Karl Storz video stylet in patients with simulated cervical fracture injuries.

Material and methods

The study, approved by the Board of Studies and the Ethical Committee of Jawaharlal Nehru Medical College and Hospital in Aligarh, involved 50 patients undergoing operative procedures under general anaesthesia. It was a prospective randomised controlled study on patients aged 20-60, weighing 30-80 kg, and classified as American Society of Anesthesiologists (ASA) Grades I and II, admitted for elective operative procedures. Patients were randomly assigned to two groups for intubation using different devices: the control group (N = 25) was intubated with the C-MAC (Mac blade) video laryngoscope (CM), and the study group (N = 25) was intubated with the Karl Storz video stylet (VS). The anaesthetic procedure involved a detailed pre-anesthetic check-up for all patients, including a medical history review, physical examination, and necessary tests based on age. Standard monitoring and pre-medication were administered uniformly. Anesthesia was induced and intubation was attempted using appropriate devices, following manual stabilisation of the neck. Parameters such as intubation attempts, time taken, failures, hemodynamic changes, and complications were recorded throughout the procedure. If intubation was unsuccessful, alternative measures were taken, and the operative procedure proceeded.

Results

The intubation success rates were compared between the two groups, CM and VS. In the CM group, all 25 patients (100%) were successfully intubated on the first attempt, while in the VS group, 23 patients (92%) were successfully intubated on the first attempt, and two patients (8%) required two attempts. The difference in the distribution of the number of attempts between the two groups was not statistically significant (p = 0.4915). The mean intubation time in the CM group was 27.24 ± 2.16 seconds, while in the VS group, the mean intubation time was significantly longer at 30.84 ± 6.81 seconds, with a statistically significant difference (p = 0.0105). Adjustment manoeuvres were required in only 4% of patients in the CM group compared to 0% in the VS group, although this difference was not statistically significant. The occurrence of blood on the device during intubation was recorded, and the distribution of patients with blood on the device among the two groups did not show a statistically significant difference (p = 0.617).

Conclusion

This study compared the effectiveness of two intubation devices. The C-MAC video laryngoscope showed a significantly higher rate of first-attempt successful intubations and required fewer attempts compared to the Karl Storz video stylet. The C-MAC also had shorter intubation times compared to the Karl Storz device. However, the Karl Storz video stylet demonstrated comparable performance to the C-MAC video laryngoscope in clinical settings, with both devices having similar safety profiles and minimal complications.

## Introduction

Maintaining or restoring ventilation is paramount in airway management, especially in cases of difficult airways, which could be life-threatening and pose challenges for anaesthesiologists [1]. The ABCDE (airway, breathing, circulation, disability, exposure) approach is essential for managing emergencies across various medical fields, ensuring prompt evaluation and care for critically ill patients. Safe anaesthetic practice prioritised securing a patent airway, preventing aspiration, and clearing tracheobronchial secretions, though complexities arose requiring careful understanding for effective management [1,2]. Tracheal intubation, a fundamental procedure in airway management, involves the insertion of a tube into the trachea to maintain a patient's airway, facilitate mechanical ventilation, and administer medications or gases directly to the lungs [3,4]. This intervention was critical in various clinical settings, from emergency medicine and anaesthesia to intensive care and surgery, where ensuring adequate oxygenation and ventilation was paramount for patient survival and recovery [5-7]. In patients with cervical spine injuries, tracheal intubation assumed even greater significance due to the unique challenges posed by the potential for spinal cord injury and instability. Cervical spine fractures, resulting from trauma or pathological conditions, could compromise the integrity of the spinal column, leading to neurological deficits, respiratory compromise, and even life-threatening complications, such as spinal cord injury [8,9]. Given the potential risks associated with traditional intubation techniques, such as direct laryngoscopy, in patients with cervical spine injuries, there was a growing emphasis on exploring alternative approaches that offered improved safety and efficacy [10]. Video-assisted techniques such as video laryngoscopy and video stylet emerged as promising alternatives that provided enhanced visualisation of the airway structures and facilitated navigation in challenging anatomical scenarios. By leveraging real-time video images, these technologies enabled clinicians to optimise the intubation process, reduce procedural complications, and improve patient outcomes, particularly in cases where cervical spine injury complicated airway management. The C-MAC video laryngoscope was the first Macintosh-type video laryngoscope. The C-MAC video laryngoscope (Karl Storz GmbH & Co. KG, Tuttlingen, Germany) contained a small camera and a light source at the distal third of the blade. The blade was connected to a portable TFT screen (7″) unit [11,12]. Different sizes of Macintosh-shaped blades were available for the C-MAC. It was a laryngoscope equipped with a miniature camera at its tip, and it provided a real-time video image of the glottic structures on a monitor, allowing clinicians to visualise the airway indirectly [13,14]. This technology offered several advantages over direct laryngoscopy, including a broader field of view, better visualisation of anatomical structures, and easier navigation of difficult airways. In patients with cervical spine fractures, video laryngoscopy offered particular benefits by reducing the need for extensive neck manipulation and providing a clearer view of the vocal cords, thereby minimising the risk of exacerbating spinal cord injury or causing further instability.

Karl Storz, a German medical instrument manufacturer, first introduced the Karl Storz video stylet. The video stylet as a category of medical devices began to emerge in the late 20th century with advancements in endoscopic technology [15]. The Karl Storz video stylet typically consisted of a slim, rigid tube with a flexible tip and a diameter ranging from 3 to 5 mL. The device was generally long enough to reach the patient's vocal cords comfortably [16]. At the distal end of the tube, there was a miniature camera or imaging sensor, accompanied by LED lights for illumination. The camera captured high-resolution images of the airway that helped visualise the intubation process. Video stylets offered advantages such as improved control and enhanced visualization of the glottic structures, particularly in patients with distorted airway anatomy or limited mouth opening. In the context of cervical spine injuries, video-stylet intubation offered distinct advantages by allowing for a more controlled and less traumatic approach to securing the airway, thereby reducing the risk of exacerbating spinal cord injury or causing vertebral dislocation. The comparative evaluation of C-MAC and Karl Storz video stylet in terms of first-attempt success rate of tracheal intubation had not been examined in any study. By conducting a prospective randomised controlled study comparing video laryngoscopy and video stylet in patients with simulated cervical spine fracture injuries, we aimed to address several key questions. Firstly, we sought to determine which technique offered superior intubation success rates and procedural outcomes, including time to intubation and rates of successful first-pass intubation. Additionally, we aimed to assess the safety profiles of both modalities by examining rates of procedural complications, such as oesophagal intubation, dental trauma, and hemodynamic instability.

Through meticulous methodology and rigorous analysis, this study endeavoured to provide evidence-based insights that had the potential to revolutionise the approach to tracheal intubation in patients with cervical spine fractures. The findings of this research held significant implications for clinical decision-making, patient care, and healthcare resource utilization in the management of patients with simulated cervical spine fracture injuries. Clinicians frequently encountered challenges in selecting the most appropriate airway management technique for these patients. By evaluating the efficacy and safety of the C-MAC video laryngoscope and the Karl Storz video stylet in achieving successful tracheal intubation on the first attempt, this study provided evidence-based guidance for clinical decision-making.

## Materials and methods

This study was conducted at Jawaharlal Nehru Medical College and Hospital, Aligarh Muslim University (JNMCH, AMU, Aligarh), involving 50 patients scheduled for operative procedures under general anaesthesia. Approval for the study protocol (IECJNMC/794) was granted by the Board of Studies, Department of Anaesthesiology Critical Care and Ethical Committee of JNMCH, AMU, Aligarh. Eligible participants, aged 20-60 years with American Society of Anesthesiologist (ASA) Grades I and II classification and weighing between 30 and 80 kg, were included after obtaining written informed consent, indicating their voluntary participation in this institutional research. Patients with anticipated difficult airways (restricted head and neck movements, thyromental distance < 6 cm), limited mouth opening (< 2.5 cm), hemodynamic or respiratory compromise, or a potentially full stomach were excluded. Randomisation using computer-generated tables allocated patients equally into two groups: the control group (N = 25) to be intubated with the C-MAC (Mac blade) video laryngoscope (CM), and the study group (N = 25) to be intubated with the Karl Storz video stylet (VS). Before the commencement of the study, proficiency with each device was ensured through a structured learning phase involving 15 insertions on mannequins and 10 insertions on patients for both devices. Patients were explained the procedure during the preoperative visit and written informed consent was taken.

Procedure

A detailed pre-anaesthetic check-up (PAC), including history, clinical examination, and routine investigations as guided by age, was carried out in all patients. After explaining the entire procedure, we obtained written informed consent in a language understood by the patient. Patients were kept nil per oral (NPO) for eight hours before surgery. Upon arrival in the OT, an 18G/20G peripheral intravenous catheter was secured. Standard multichannel monitoring was used throughout the procedure, including non-invasive blood pressure (NIBP), electrocardiograph (ECG), pulse oximetry (SPO_2_), and end-tidal carbon dioxide (ETCO2). Uniform premedication was done with Inj. glycopyrrolate 0.2 mg IV, Inj. midazolam 0.03 mg/kg IV, Inj. dexamethasone 0.1 mg/kg IV, and Inj. fentanyl 2.0 mcg/kg IV. After pre-oxygenation with 100% oxygen for three minutes, anaesthesia was induced with Inj. propofol 2.0 mg/kg IV. After adequate muscle relaxation with Inj. succinylcholine 1.5 mg/kg IV, manual inline stabilisation (MILS) was applied by trained personnel, and the patients were intubated with the device depending on the group to which the patient was assigned (group CM/VS). A maximum of three attempts were allowed with or without external laryngeal manipulation (OELM). Successful intubation was confirmed by the ability to achieve a tidal volume of at least 7-8 mL/kg, five-point auscultation, and a square wave capnogram. The appearance of the first square waveform was taken as the endpoint of the study. If intubation could not be performed within three attempts or within 120 seconds in each group, the application of MILS was removed, head and neck flexion-extension was allowed to intubate the patient with the same device, or a second-generation supraglottic airway device was inserted. The operative procedure was allowed to proceed. The case was considered a failure. The following parameters were recorded: number of attempts, total time taken for intubation, number of failures, number of patients requiring external laryngeal manipulation, hemodynamic changes, and any complications.

Device and description

C-MAC Video Laryngoscope

**Figure 1:**
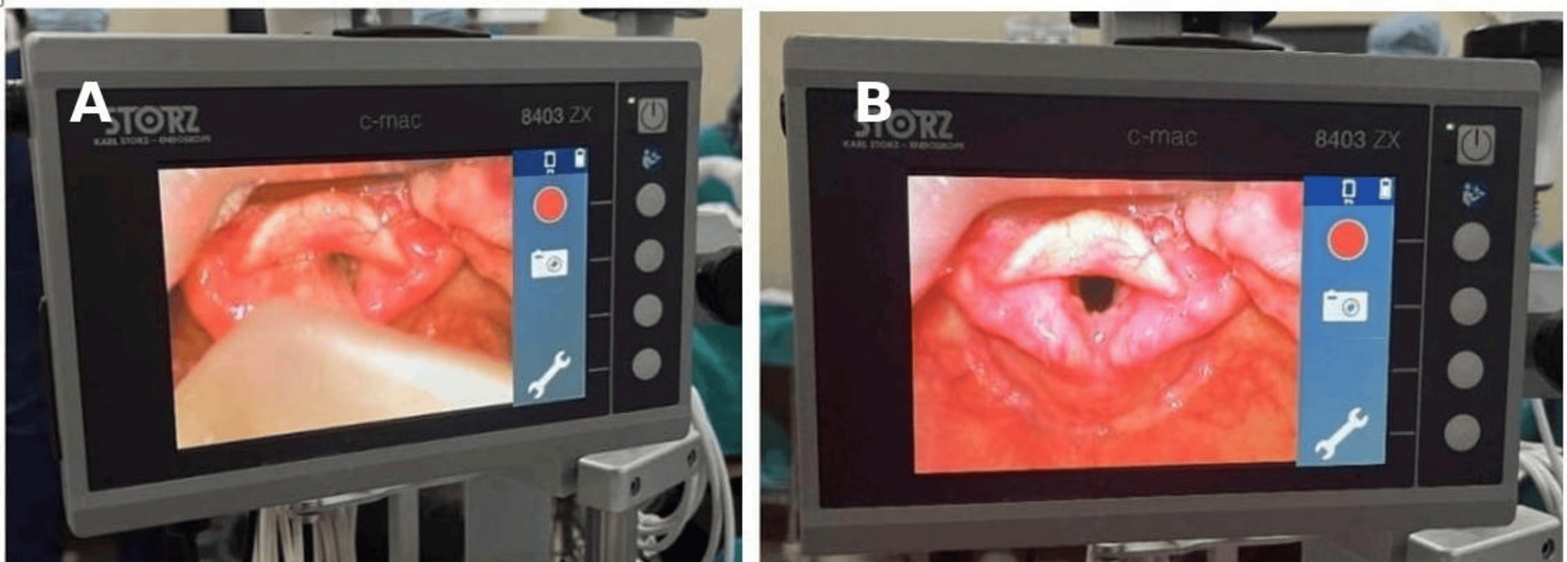
Two side-by-side screens from the C-MAC video laryngoscope showing the glottic view Screen A (left screen): This screen shows a close-up view of the laryngeal structures, including the vocal cords and surrounding tissues. The vocal cords appear to be in a partially open position. The interface includes icons for recording (red circle), taking a photo (camera icon), and accessing settings (wrench icon). Screen B (right screen): This screen also displays a close-up view of the larynx, similar to Screen A, but the vocal cords appear slightly more open. The interface has the same icons as Screen A for recording, taking photos, and accessing settings.

The C-MAC video laryngoscope, introduced by Karl Storz GmbH & Co. KG (Tuttlingen, Germany) in 1999, represents a significant advancement in airway management technology. This device integrates a high-resolution color video camera into a traditional laryngoscope handle, paired with a Macintosh blade. The system features a combined image and light bundle that runs through a metal guide tube, positioned 40 mm from the blade tip to optimize visualisation. The camera cable connects to a control unit, while the light cable is linked to a light source, facilitating seamless operation. The C-MAC is mounted on a compact, mobile cart equipped with an 8-inch monitor on a swivel arm, which is conveniently placed on the patient’s left side for optimal viewing. One of its standout features is the ability to perform both direct and video laryngoscopy using the same device, enhancing its versatility, especially in emergency intubation scenarios. The advanced visualisation capabilities of the C-MAC contribute to improved laryngeal views and higher success rates in intubations, particularly in patients with anticipated or confirmed difficult airways. As illustrated in Figure 1, the device provides a clear view of the larynx, demonstrating its effectiveness in achieving optimal intubation conditions.

Karl Storz Video Stylet

**Figure 2:**
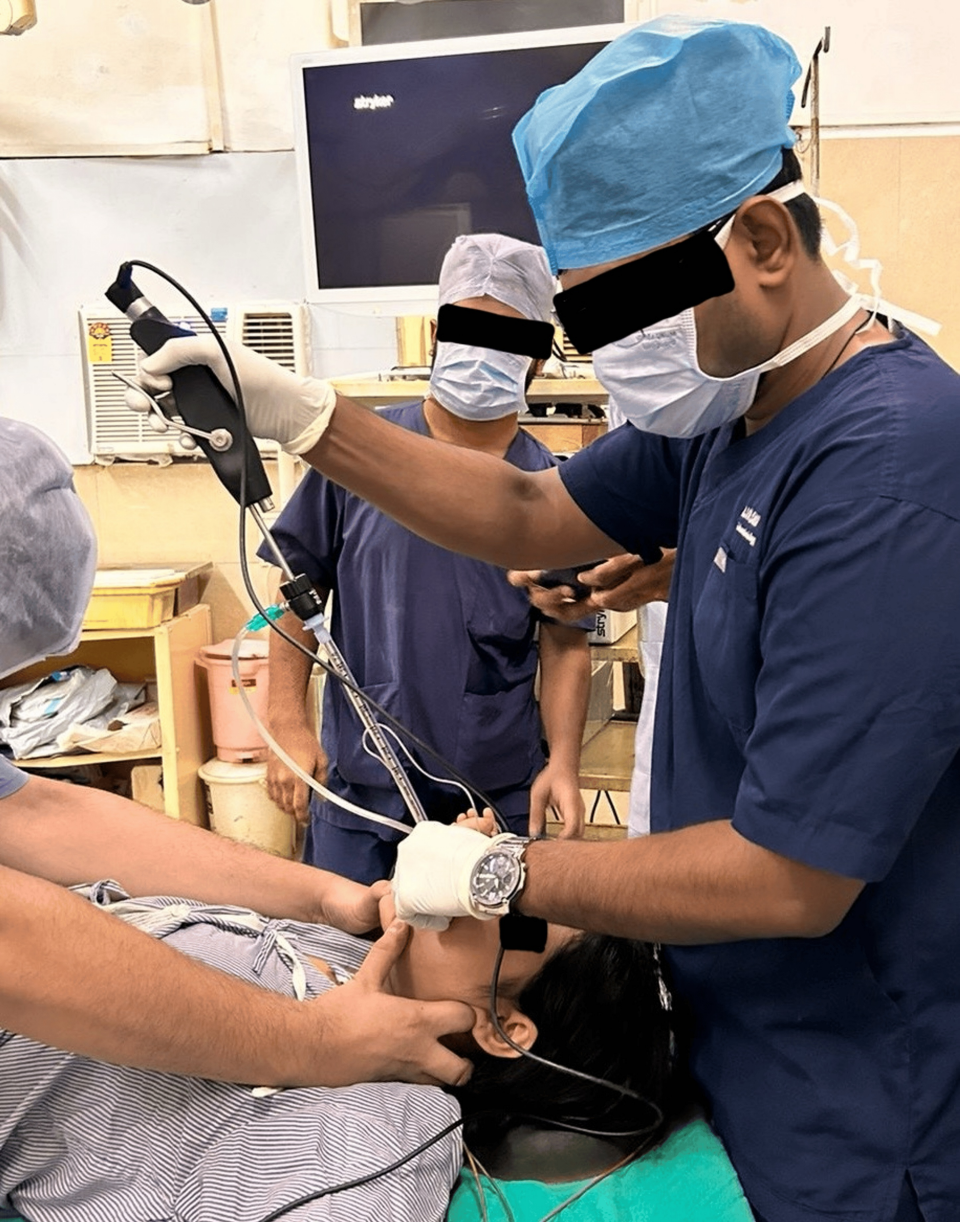
Intubation attempt using the Karl Storz video stylet The figure shows an intubation attempt using the Karl Storz video stylet. The endotracheal tube is railroaded into the stylet and introduced between incisors (midline approach).

**Figure 3:**
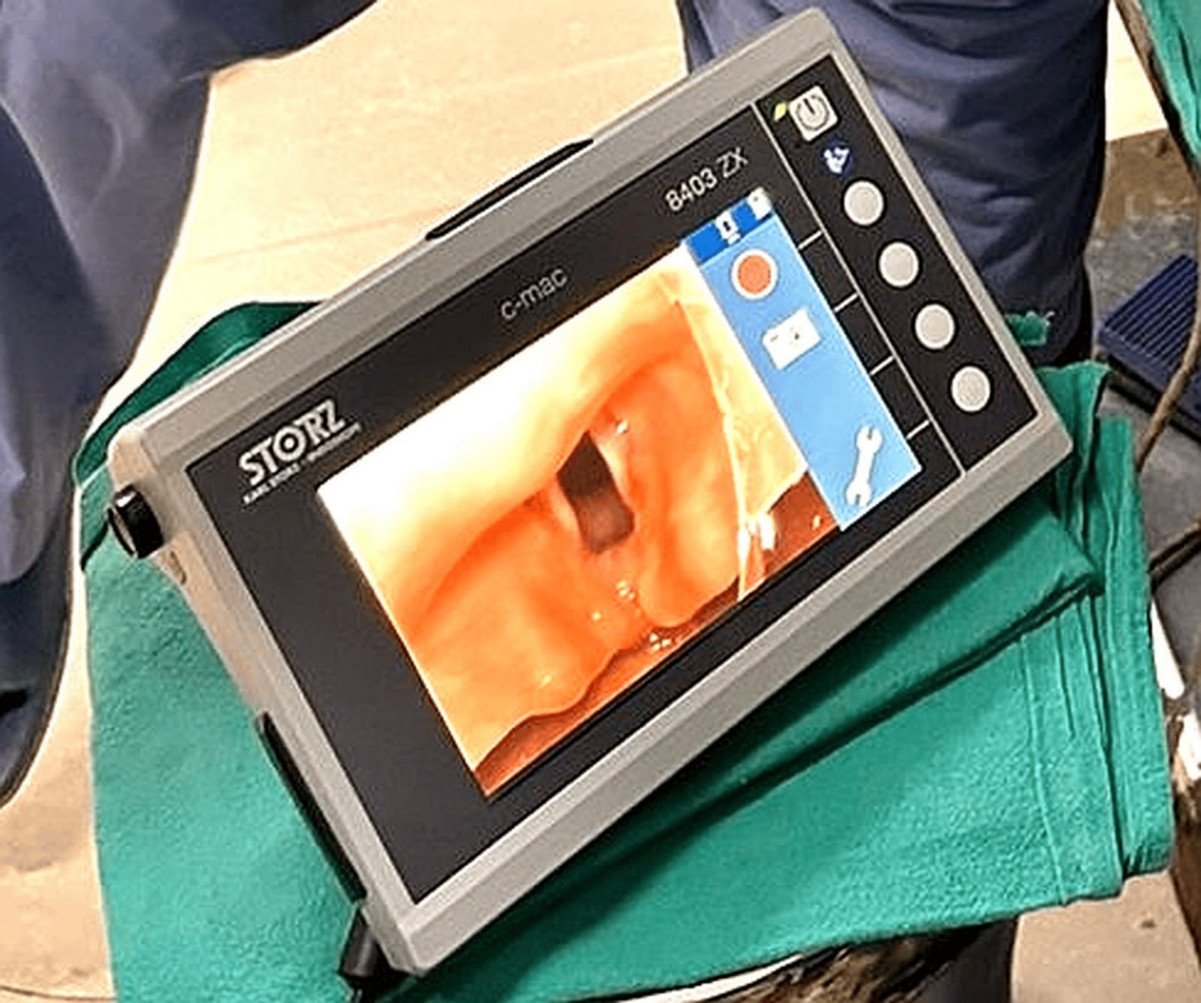
Glottic view with the Karl Storz video stylet The screen shows a close-up view of the laryngeal structures, including the vocal cords and surrounding tissues. The crescent shape of the railroaded endotracheal tube is visible at the right side of the glottic opening.

The C-MAC® VS represents an advanced evolution from the traditional retromolar intubation endoscope, offering a hybrid of rigid and flexible intubation techniques. Designed with a sheath and a deflectable tip, it is particularly advantageous in bariatric surgery for patients with limited mouth opening or cervical spine issues. The device features a lever with passive return, allowing for easy adaptation to various anatomical conditions, and deflection up to 60° with a loaded endotracheal tube (ETT). The high-resolution CMOS chip delivers clear images in a 4:3 format without the Moiré effect, enhancing visualisation during intubation. Continuous oxygen flow is facilitated through a tube adaptor, extending the time window for intubation. The C-MAC® VS can be reprocessed up to 65°C and includes a BlueButton for documentation, offering innovative multifunctionality. Its Plug & Play compatibility with the universal C-MAC® system interface and watertight construction (IPX8) ensure reliability and ease of use. Figure 2 illustrates an intubation attempt using the Karl Storz video stylet, while Figure 3 provides a clear view of the glottis through the video stylet, demonstrating the system's effectiveness in providing optimal intubation conditions.

Statistical analysis

The data entry was conducted using Excel (Microsoft® Corp., Redmond, WA) spreadsheets, followed by the final analysis using Statistical Product and Service Solutions (SPSS, version 25.0; IBM SPSS Statistics for Windows, Armonk, NY) software. Categorical variables were presented as numbers and percentages (%), while continuous variables were reported as mean ± standard deviation (SD) and median values. Statistical significance was determined using a p-value less than 0.05. For qualitative variables, comparisons were analyzed using the chi-square test, with Fisher’s exact test utilized when any expected cell value was less than 5. Quantitative variables were analyzed using ANOVA, supplemented by a post-hoc test with Bonferroni correction for multiple comparisons. Data related to ease of intubation and post-operative complications, such as blood on the device and sore throat, were evaluated using Fisher’s exact test. The time taken for intubation was assessed using ANOVA. These statistical methods were employed to rigorously analyze and interpret the study findings.

## Results

Parameters

Demographic Data

The demographic characteristics of the patients in the CM group and VS group were comparable. The mean age in the CM group was 36 ± 9.2 years, while in the VS group, it was 36 ± 9.9 years (p = 0.985). The gender distribution showed that 32% of the CM group were male and 68% were female, compared to 24% male and 76% female in the VS group (p = 0.529). The mean weight was 58.6 ± 8.98 kg in the CM group and 59.8 ± 8.71 kg in the VS group (p = 0.627) (Table 1).

**Table 1:** Distribution of patients according to various parameters such as demographic data, age groups, gender, Mallampati grading, number of attempts for intubation, success of intubation, mean duration of time taken for intubation, number of adjustment manoeuvres, blood on device (Bod), and postoperative sore throat in both the groups

Parameter	Sub-parameter	CM Group (n=25)	VS Group (n=25)	p-value
Demographic Data	Age (years) mean ± SD	36 ± 9.2	36 ± 9.9	0.985
	Male	8 (32%)	6 (24%)	0.529
	Female	17 (68%)	19 (76%)	
	Weight (kg) mean ± SD	58.6 ± 8.98	59.8 ± 8.71	0.627
Distribution by Age Group	20-30	6 (24%)	5 (20%)	0.954
	31-40	8 (32%)	9 (36%)	
	41-50	7 (28%)	6 (24%)	
	51-60	4 (16%)	5 (20%)	
	Total	25 (100%)	25 (100%)	
Gender Distribution	Male	8 (32%)	6 (24%)	0.529
	Female	17 (68%)	19 (76%)	
	Total	25 (100%)	25 (100%)	
Mallampati Grading	Grade 1	1 (4%)	0 (0%)	0.357
	Grade 2	6 (24%)	6 (24%)	
	Grade 3	16 (64%)	18 (72%)	
	Grade 4	2 (8%)	1 (4%)	
Number of Attempts	1 Attempt	25 (100%)	23 (92%)	0.4915
	2 Attempts	0 (0%)	2 (8%)	
Success of Intubation	Success	25 (100%)	25 (100%)	
	Fail	0 (0%)	0 (0%)	
	Total	25	25	
Intubation Time	Mean ± SD (seconds)	27.24 ± 2.16	30.84 ± 6.81	0.0105
Adjustment Manoeuvres	No Adjustment	24 (98%)	25 (100%)	1.00
	Required Adjustment	1 (4%)	0 (0%)	
Blood on Device	Yes	1 (4%)	3 (12%)	0.617
	No	24 (96%)	22 (88%)	
Sore Throat	Yes	3 (12%)	2 (8%)	0.672
	No	22 (88%)	23 (92%)	

Age Distribution

Patients were distributed across various age groups. In the CM group, 24% were aged 20-30, 32% were aged 31-40, 28% were aged 41-50, and 16% were aged 51-60. In the VS group, 20% were aged 20-30, 36% were aged 31-40, 24% were aged 41-50, and 20% were aged 51-60. The distribution across age groups did not differ significantly between the two groups (p = 0.954) (Table 1).

Gender Distribution

The gender distribution was 32% male and 68% female in the CM group, compared to 24% male and 76% female in the VS group. This difference was not statistically significant (p = 0.529) (Table 1).

Mallampati Grading

The distribution of patients according to Mallampati grading showed no significant differences between the groups. In the CM group, 4% were classified as Grade 1, 24% as Grade 2, 64% as Grade 3, and 8% as Grade 4. In the VS group, 0% were Grade 1, 24% were Grade 2, 72% were Grade 3, and 4% were Grade 4 (p = 0.357) (Table 1).

Number of Intubation Attempts

All patients in the CM group were successfully intubated on the first attempt (100%), while 92% of patients in the VS group were successfully intubated on the first attempt, and 8% required two attempts. This difference was not statistically significant (p = 0.4915) (Table 1).

Success of Intubation

Both groups achieved a 100% success rate for intubation (p = not applicable) (Table 1).

Duration of Intubation

The mean intubation time was significantly shorter in the CM group at 27.24 ± 2.16 seconds, compared to 30.84 ± 6.81 seconds in the VS group (p = 0.0105) (Table 1).

Adjustment Manoeuvres

Adjustment manoeuvres were required in 4% of patients in the CM group and in 0% of patients in the VS group. This difference was not statistically significant (p = 1.00) (Table 1).

Blood on Device

The occurrence of blood on the device during intubation was 4% in the CM group and 12% in the VS group, with no statistically significant difference (p = 0.617) (Table 1).

Sore Throat

Postoperative sore throat was reported in 12% of patients in the CM group and 8% in the VS group, which was not statistically significant (p = 0.672) (Table 1).

The C-MAC video laryngoscope showed a significantly higher rate of first-attempt successful intubations and required fewer attempts compared to the Karl Storz video stylet. The C-MAC also had shorter intubation times compared to the Karl Storz device.

## Discussion

The present study aimed to compare the performance of the C-MAC video laryngoscope (CM) and the Karl Storz video stylet (VS) for tracheal intubation in patients with simulated cervical fracture injuries. The primary outcome was the first-attempt success rate of tracheal intubation, while secondary outcomes included the time required for successful intubation, the number of attempts needed, the necessity for adjustment manoeuvres (such as external laryngeal manipulation or stylet adjustments), and the incidence of postoperative airway complications.

Demographic data

The demographic data, including age, sex, and weight, were comparable between the two groups, indicating that the study population had been well-matched and that these factors had not influenced the results.

First-attempt success rate

The results revealed a higher first-attempt success rate for tracheal intubation in the CM group (100%) compared to the VS group (92%). However, this difference was not statistically significant (p = 0.4915). This finding suggested that both the C-MAC video laryngoscope and the Karl Storz video stylet were effective tools for intubation, providing high success rates even in patients with simulated cervical fracture injury. Despite the slightly higher success rate in the CM group, the similar performance between the two methods indicated that either device could be reliably used in clinical practice.

Comparing these results with those of Aziz et al. [17], it became evident that video laryngoscopy demonstrated consistent advantages over conventional direct laryngoscopy in various clinical scenarios. Aziz et al. reported a higher first-attempt success rate with video laryngoscopy (93%) compared to direct laryngoscopy (84%) (p = 0.026). This aligned with our findings, where the C-MAC group exhibited a significantly higher first-attempt success rate (92%) compared to the VS group (48%) (p < 0.001). Furthermore, both studies noted improved Cormack-Lehane laryngeal views and reduced reliance on adjunctive manoeuvres with video laryngoscopy, underscoring its efficacy in challenging intubation scenarios. However, Aziz et al. [17] did not find a significant difference in complication rates between video laryngoscopy and direct laryngoscopy (p = 0.146). Our study did not directly compare complication rates between the C-MAC and VS groups. Despite this disparity, the collective evidence supported the notion that video laryngoscopy, including both the C-MAC and Karl Storz video stylet, offered tangible benefits over traditional methods, particularly in cases of simulated cervical fracture injury where optimal glottic visualization was crucial for successful intubation.

Our results aligned with those of Yoon et al. [18], who also investigated the first-attempt success rate of tracheal intubation using different devices. In their study, the first-attempt success rate was significantly higher in group M compared to group O, with values of 92.3% and 81.0%, respectively (p = 0.002). Additionally, the intubation time was significantly shorter in group M compared to group O, with mean differences of 13.5 seconds (p = 0.001). Notably, group O included five patients whose tracheas could not be intubated within three attempts due to anatomical factors, necessitating alternative methods for successful intubation.

The consistency in findings across both studies underscored the efficacy of certain devices, such as the C-MAC video laryngoscope and the device used in group M in the Yoon et al. [18] study, in improving first-attempt success rates and reducing intubation time. These results highlighted the importance of device selection in optimizing airway management, particularly in challenging clinical scenarios. Despite differences in patient populations and study methodologies, the robustness of the findings supported the clinical relevance of these devices in enhancing procedural success and patient safety during tracheal intubation.

Time for successful intubation

The analysis of the mean duration of time taken for successful intubation revealed significant differences between the CM and VS groups. The mean intubation time in the CM group was 27.24 ± 2.16 seconds, while the VS group had a significantly longer mean intubation time of 30.84 ± 6.81 seconds (p = 0.0105). This statistically significant difference indicated that the C-MAC video laryngoscope allowed for quicker intubation compared to the Karl Storz video stylet.

Our study, alongside findings from Ahmed et al. [19], supported the efficacy of video laryngoscopy, particularly with the C-MAC device, in facilitating tracheal intubation. Both studies showed significantly shorter intubation times with the C-MAC compared to alternative methods. While success rates were similar across groups, advantages in glottic visualization and ease of intubation were apparent with the C-MAC. Furthermore, Ahmed et al. [19] noted reduced hemodynamic perturbations with C-MAC, highlighting enhanced patient safety. These findings underscored the growing preference for video laryngoscopy in optimizing intubation processes and minimizing complications.

Comparing the findings of Zhang et al. [16] with our study shed light on the efficacy of different intubation techniques and devices in clinical settings. In Zhang et al. [16], the average duration for intubation varied across groups, with the VS group (video stylet), demonstrating the shortest intubation time of 26.88 ± 4.51 seconds, followed by the VL group (video laryngoscope) at 44.56 ± 4.42 seconds, and the FV group at 95.20 ± 4.01 seconds. The results of this study were contrary to our results, but they used a different type of curved rigid video stylet, instead of the rigid straight stylet that we used in our study. Interestingly, despite differences in intubation time, there were no significant variations in the occurrence of intubation-related adverse reactions among the groups. Zhang et al.'s [16] findings underscored the advantage of VL over FV in terms of intubation duration, with VS demonstrating the shortest intubation time. They also used a different type of video stylet, which was curved and rigid compared to the straight stylet by Karl Storz.

Number of attempts and adjustment manoeuvres

The comparison of the number of attempts for successful intubation between the CM and VS groups revealed important insights into the performance of these intubation methods. In the CM group, all 25 patients (100%) were successfully intubated on the first attempt. In contrast, in the VS group, 23 patients (92%) were successfully intubated on the first attempt, with two patients (8%) requiring a second attempt. Despite the perfect first-attempt success rate in the CM group, the difference in the distribution of the number of attempts between the two groups was not statistically significant (p = 0.4915). This suggested that both methods were highly effective for achieving first-attempt intubation.

Amaniti et al. [20] reported that, out of 176 patients, 40 required more than one attempt for successful intubation. Within this subset, 12 cases necessitated a transition from the classic insertion of the video laryngoscope blade with tongue sweeping to midline insertion. This manoeuvre, while not directly comparable to the adjustment manoeuvres observed in our study, highlighted the dynamic nature of intubation procedures and the need for adaptability among clinicians.

Postoperative airway complications

The incidence of postoperative airway complications, including blood on the device and sore throat, was similar between the two groups. The difference in the occurrence of blood on the device (CM: 4%, VS: 12%, p = 0.617) and sore throat (CM: 12%, VS: 8%, p = 0.672) was not statistically significant.

In contrast, the study by Yoon al. [18] revealed consistent findings with the previous study regarding postoperative airway complications. The incidences of postoperative sore throat and hoarseness at one hour (20.8% vs 25.0%; p = 0.400 and 8.7% vs 7.1%; p = 0.687, respectively) and 24 hours after surgery (10.9% vs 14.7%; p = 0.359 and 4.4% vs 3.8%; p = 0.991, respectively) were comparable between the two groups. These results mirrored the findings of the first study, indicating no significant difference in the occurrence of these complications between the intervention and control groups. It was noteworthy that the overall incidence of postoperative neurologic complications was low (6.3%) in Yoon et al.'s [18] study, with no significant difference observed between the two groups (7.7% vs 4.9%; risk difference (95% confidence interval), 2.8% (−2.3 to 8.1); p = 0.382). These results reinforced the safety profile of both interventions concerning neurologic outcomes.

Kapadia et al. [21] showed no statistically significant difference in the incidence of postoperative sore throat between patients in the CL and VL groups across multiple time points (first, 12th, and 24th hours postoperatively). Despite slight variations in the percentages, none of these differences reached statistical significance, indicating a similar trend in postoperative sore throat occurrence between the two groups over the postoperative period.

Overall, the results of this study suggested that the C-MAC video laryngoscope might have been a superior option compared to the Karl Storz video stylet for tracheal intubation in patients with simulated cervical fracture injury. The higher first-attempt success rate and shorter intubation time observed with the C-MAC video laryngoscope could be attributed to several factors, including better glottic visualisation, ease of use, and familiarity with the device among the anesthesiologists performing the intubations. The difficulties faced with Karl Storz's video stylet included the manoeuvrability of the straight rigid stylet, which made alignment of oral, pharyngeal, and laryngeal axes comparatively difficult. The flexible tip of the VS was useful during the visualisation of vocal cords and epiglottis, but sliding the endotracheal tube through the device and vocal cords was potentially difficult. Armoured or flexometallic endotracheal tubes were better compatible with VS. For a better view using VS, the epiglottis was lifted by jaw thrust, which had to be done in all cases using VS.

It was important to note that the study was conducted in a controlled setting with simulated cervical fracture injury, and the results might not have been directly applicable to real-life situations involving acute cervical spine injuries. Additionally, the relatively small sample size and potential for operator bias could have been limitations of the study.

Future research could have explored the performance of these devices in actual patients with cervical spine injuries, as well as compared them with other airway management techniques, such as flexible fiberoptic intubation or video-assisted intubation devices. Furthermore, the impact of operator experience and training on the performance of these devices could have been an area of interest for future investigations.

The findings suggested that the C-MAC video laryngoscope might have offered advantages in terms of faster intubation times and higher first-attempt success rates, potentially translating to better patient outcomes in clinical settings. However, both devices exhibited similar safety profiles, making them viable options for intubation with comparable complication rates.

Future research should have focused on larger sample sizes and diverse patient populations to validate these findings further. Additionally, exploring the learning curve associated with each device and the impact of operator experience on outcomes could have provided deeper insights into optimizing intubation practices.

Limitations

This study encountered several limitations. Firstly, it was conducted at a single center with a limited number of patients, which might have restricted the generalizability of the findings. Secondly, the patient cohort included individuals undergoing heterogeneous surgical procedures, introducing variability in clinical contexts. Notably, the simulation of cervical fracture injury using manual inline stabilization instead of actual cervical fractures could have influenced outcomes, particularly in emergency care settings where conditions might differ. The video stylet might have performed better in patients with restricted mouth opening. Therefore, further research was warranted to validate and extend the findings of this investigation, potentially involving larger and more diverse patient populations across multiple centers to enhance the robustness and applicability of the results.

## Conclusions

In this study, we compared the C-MAC video laryngoscope and the Karl Storz video stylet for intubation. Both devices demonstrated similar effectiveness and safety across different patient groups and procedural outcomes. The CM device showed slightly higher first-attempt success rates and significantly shorter intubation times compared to the VS device. However, both devices successfully performed intubations with few complications. These findings suggest that the CM device's efficiency may offer practical benefits in clinical settings, particularly in critical care scenarios where quick intervention is essential. Conversely, the video stylet may be more suitable for patients with limited mouth opening. Future research should explore these devices in larger and more diverse patient populations to validate these results and refine their use in enhancing patient care and safety.
